# Lifelong nnU-Net: a framework for standardized medical continual learning

**DOI:** 10.1038/s41598-023-34484-2

**Published:** 2023-06-09

**Authors:** Camila González, Amin Ranem, Daniel Pinto dos Santos, Ahmed Othman, Anirban Mukhopadhyay

**Affiliations:** 1 Technical University of Darmstadt, Karolinenpl. 5, 64289 Darmstadt, Germany; 2 University Hospital Cologne, Kerpener Str. 62, 50937 Cologne, Germany; 3 University Hospital Frankfurt, Theodor-Stern-Kai 7, 60590 Frankfurt, Germany; 4 University Medical Center Mainz, Langenbeckstraße 1, 55131 Mainz, Germany

**Keywords:** Translational research, Medical imaging, Computer science

## Abstract

As the enthusiasm surrounding Deep Learning grows, both medical practitioners and regulatory bodies are exploring ways to safely introduce image segmentation in clinical practice. One frontier to overcome when translating promising research into the clinical open world is the shift from *static* to *continual* learning. Continual learning, the practice of training models throughout their lifecycle, is seeing growing interest but is still in its infancy in healthcare. We present *Lifelong nnU-Net*, a standardized framework that places continual segmentation at the hands of researchers and clinicians. Built on top of the nnU-Net—widely regarded as the best-performing segmenter for multiple medical applications—and equipped with all necessary modules for training and testing models sequentially, we ensure broad applicability and lower the barrier to evaluating new methods in a continual fashion. Our benchmark results across three medical segmentation use cases and five continual learning methods give a comprehensive outlook on the current state of the field and signify a first reproducible benchmark.

## Introduction

Deep Learning methods for medical use cases continue to be evaluated in a *static* setting, where all available data is shuffled and the model is tested on a subset of in-distribution samples. This stands on the unrealistic assumptions that (a) all training data is available in a central location, and (b) the acquisition conditions do not change over time after clinical deployment^1^. Evaluating in this manner creates a considerable gap between the reported performance of new methods and their usability in practice^2–4^, which hinders the vital deployment of lifelong learning agents in dynamic clinical environments^5^.

**Figure 1:**
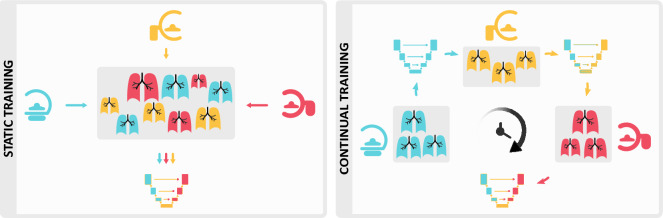
In a static setting (left), all training data is brought together. Continual settings (right) consider the time of acquisition and train the model sequentially.

*Continual learning* does not neglect the temporal dimension of the data and trains models in a sequential fashion, as illustrated in Fig. 1. The goal hereby is to adapt to new environments without losing performance on previously observed training conditions and subject groups. Distributed *federated learning* methods have been explored in multi-clinical settings and also do not require sharing data between institutions^6,7^. However, they neither address *temporal restrictions on data availability* nor provide a framework for agents that continuously adapt to shifting population dynamics. Continual learning in healthcare, which tackles these concerns, is receiving growing enthusiasm^8–11^ and regulatory procedures are being actively debated^5,12,13^. Currently, re-approval is required each time a model is adapted during deployment, but there are initiatives from both the FDA and European Commission for a *lifecycle regulatory protocol* that allows the use of continuously adapting algorithms^14^. These pursuits may lead us to the rare situation where the regulatory guidelines are in place while the technology is still in its infancy.

Technical literature of continual learning for simpler computer vision tasks is plagued by controversies about the lack of a standardized evaluation setup^15–17^. Recently, the *Avalanche*^18^ project has emerged as a solution to this problem for continual classification by providing a unified code base. The field is not as mature for continual *segmentation*, which assigns a label to each pixel in the image and is arguably the primary AI task in the clinical domain. Though more work has been done in recent years^8,10,19–23^, it neither (1) builds on top of high-performing segmentation pipelines nor (2) examines how popular methods transfer to image segmentation for multiple open-source benchmarks.

In this work, we present *Lifelong nnU-Net*, a standardized framework for training and evaluating segmentation models in continual settings. We build our code on top of the *nnU-Net* pipeline, which is widely popular and state-of-the-art for 33 medical segmentation tasks—and competitive for twenty additional ones—across 11 international biomedical segmentation challenges^24^. This ensures the high usability and performance of our extended framework. Our contributions are:

- The introduction of an open-source continual learning framework built on top of the *nnU-Net*
- A performance and run-time comparison for training sequentially under different settings, and
- Open-source implementations for *five continual learning methods*, allowing the fast evaluation of the state-of-the-art and accelerating the development of new approaches.

Our experiments on publicly available data for three different segmentation problems show that:

- None of the explored continual learning methods consistently achieve *positive backward transfer* for segmentation, exhibiting the need for new solutions,
- In accordance with previous research, rehearsal-based methods display the least amount of forgetting while maintaining model plasticity, and
- The practice of maintaining task-specific heads, common in continual learning literature, is only minimally relevant for segmentation.

The goal of *Lifelong nnU-Net* is to ensure high technical standards and reproducible results while the community is translating continual learning to medical image segmentation. By *releasing our code and trained models* for open-source datasets, we establish a benchmark for evaluating future continual learning methods on segmentation models.

## Results

We start this section by examining the results of training models statically with one dataset. Afterward, we explore sequential learning and five popular continual learning strategies: *Rehearsal*, *Elastic Weight Consolidation*^25^ (EWC), *Learning without Forgetting*^26^ (LwF), *Riemannian Walk*^27^ (RW) and *Modeling the Background*^28^ (MiB). We hereby regard the datasets of each anatomy (hippocampus, prostate, or heart) as *n* tasks $\mathscr {T}_1$, ..., $\mathscr {T}_n$ and train the model of each use case sequentially with all respective tasks.

We quantify segmentation performance with the Dice coefficient and report backward transfer (BWT), which measures the degree of forgetting older tasks, and forward transfer (FWT), which assesses the ability to learn new knowledge.

Finally, we analyze the difference between using single- versus multi-head architectures, briefly illustrate the importance of task orderings and provide a summary of our training times.

**Table 1:** Continual learning performance as Dice coefficient.

	Prostate				Hippocampus		
	UCL	I2CVB	ISBI	DecathProst	HarP	Dryad	DecathHip
Static	70.91 (± 6.02)	93.05 (± 0.29)	92.27 (± 0.26)	91.90 (± 0.36)	90.48 (± 1.71)	94.12 (± 0.05)	93.99 (± 0.45)
Seq.	85.16 (± 1.24)	21.04 (± 5.63)	93.09 (± 0.36)	**91.91** (± **0.38)**	20.20 (± 5.55)	57.19 (± 1.02)	90.92 (± 1.08)
EWC	**86.87** (± **0.49)**	58.53 (± 4.73)	88.43 (± 0.61)	87.79 (± 0.83)	88.01 (± 3.47)	86.09 (± 0.59)	31.93 (± 6.09)
LwF	85.30 (± 0.82)	22.89 (± 4.82)	92.37 (± 0.36)	91.48 (± 0.33)	3.90 (± 1.97)	46.00 (± 1.62)	90.85 (± 1.08)
MiB	86.31 (± 0.62)	48.87 (± 6.55)	92.96 (± 0.39)	92.11 (± 0.27)	82.45 (± 2.94)	85.27 (± 0.32)	20.75 (± 6.99)
RW	84.08 (± 1.66)	26.51 (± 6.13)	93.18 (± 0.32)	92.07 (± 0.41)	7.33 (± 3.77)	34.87 (± 1.86)	91.07 (± 1.03)
Reh.	85.94 (± 0.76)	**90.64** (± **0.77)**	**93.39** (± **0.28)**	91.55 (± 0.34)	**88.17** (± **3.63)**	**92.07** (± **0.15)**	**91.16** (± **1.17)**

The first row shows the upper bound of training a model statically with all training data of the respective anatomy. We then see the performance of sequential training with and without (*Seq.*) several continual learning strategies (*EWC*, *LwF*, *Reh*., *MiB* and *RW*). The Dice performance is reported of the final model (after training with all tasks). The best-performing values are in **[bold]**.

**Table 2:** Continual learning performance on the cardiac use case as Dice coefficient.

	Siemens			Philips		
	LV	MI	RV	LV	MI	RV
Seq.	80.5 (± 5.0)	68.5 (± 3.8)	64.8 (± 10.1)	**96.1** (± **0.5)**	**87.0** (± **0.6)**	**93.2** (± **1.0)**
EWC $\lambda = 0.1$	**95.2** (± **0.7)**	**85.1** (± **1.2)**	90.3 (± 1.5)	94.1 (± 0.4)	81.5 (± 1.6)	88.9 (± 1.3)
EWC $\lambda = 0.2$	95.4 (± 0.7)	**85.1** (± **1.2)**	89.9 (± 1.6)	94.5 (± 0.3)	81.7 (± 1.4)	89.9 (± 0.8)
EWC $\lambda = 0.4$	92.9 (± 2.7)	83.2 (± 2.3)	88.0 (± 2.5)	93.6 (± 1.0)	82.0 (± 1.8)	87.0 (± 3.2)
LwF $T = 2$	74.2 (± 7.8)	64.0 (± 4.7)	60.6 (± 10.7)	96.0 (± 0.7)	86.8 (± 0.8)	92.2 (± 1.3)
LwF $T = 5$	69.3 (± 11.4)	61.2 (± 9.5)	54.7 (± 11.1)	96.0 (± 0.3)	85.4 (± 0.8)	91.3 (± 0.7)
LwF $T = 10$	74.7 (± 9.7)	50.3 (± 6.9)	61.7 (± 11.2)	**96.1** (± **0.3)**	85.8 (± 0.6)	92.3 (± 0.8)
MiB $\alpha =0.8$	94.5 (± 1.2)	**85.1** (± **1.3)**	**90.5** (± **1.6)**	94.9 (± 0.2)	83.3 (± 1.4)	90.6 (± 0.8)
MiB $\alpha =0.9$	91.5 (± 2.0)	80.3 (± 1.7)	80.5 (± 5.3)	95.9 (± 0.6)	86.7 (± 0.9)	92.4 (± 1.5)
MiB $\alpha =1.0$	92.7 (± 1.4)	84.1 (± 1.2)	88.4 (± 1.6)	94.5 (± 0.3)	83.0 (± 1.3)	89.9 (± 1.0)
RW $\lambda = 0.4$	82.8 (± 5.8)	71.0 (± 3.5)	67.3 (± 9.2)	**96.1** (± **0.6)**	86.7 (± 0.7)	93.1 (± 1.2)
RW $\lambda = 0.8$	74.8 (± 12.5)	67.4 (± 9.3)	69.6 (± 8.7)	**96.1** (± **0.4)**	85.8 (± 0.7)	91.6 (± 0.5)
RW $\lambda = 1.0$	74.8 (± 12.4)	65.4 (± 9.7)	71.1 (± 8.5)	96.0 (± 0.3)	85.4 (± 0.7)	91.7 (± 0.6)
Reh.	93.9 (± 0.9)	83.6 (± 1.2)	84.5 (± 4.5)	96.0 (± 0.7)	86.6 (± 1.0)	92.9 (± 1.3)

We report the results of the last model state (after training with both tasks) for sequential training, four privacy-preserving continual learning methodologies, each for three different hyperparameter settings, and training with rehearsal. The best-performing values are in **[bold]**.

### Static results and inter-task performance

**Figure 2:**
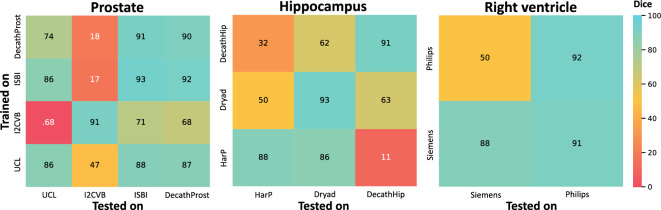
Performance of models trained independently solely on one dataset. On the (lower left to upper right) diagonal we find the Dice coefficient of evaluating models on the test cases of the dataset used for training. In the remaining cells, we see how these models transfer to other datasets. For the cardiac data, we report the right ventricle segmentation performance.

To put continual learning results into context, we first observe the performance of independent models trained solely on one dataset. These are illustrated in Fig. 2. On the diagonal from the lower left to the upper right corner, we see static evaluations on in-distribution data. In this setting, all models achieve at least an 86% Dice.

The inter-task matrices also allow us to see how effectively each model performs on out-of-distribution data. These differences in performance are due to both the inherent dissimilarity between datasets in terms of acquisition and patient population and to model robustness caused by larger and more diverse training data. The assumption is that *if a model trained on* $\mathscr {T}_1$ *is later trained on* $\mathscr {T}_2$, *the amount of forgetting for* $\mathscr {T}_1$ *will be lower the more similar the data distribution and the higher the initial performance of the model on* $\mathscr {T}_2$.

For prostate segmentation (first heatmap), *I2CVB* is a clear outlier. In the case of hippocampus, the model trained on *HarP* performs worse on *DecathHip* and the other way around. While the *HarP* model achieves a 86% Dice on *Dryad*, the *Dryad* model only reaches 50% on *HarP*. This is likely due to the much larger size of *HarP* (see Table 4). In the case of right ventricle segmentation, the model trained on *Siemens* performs well on *Philips*, but the *Philips* model only reaches a 50% Dice on the Siemens data, likely resulting for less variation among the training cases.

### Continual learning methods

Next, we inspect the performance when models are trained in a sequential fashion, summarized in Table 1 for the prostate and hippocampus anatomies and in Table 2 for cardiac. In the first row, we report the upper bound of a static model trained with all shuffled training data from the respective anatomy. The following row shows the result of training a model sequentially in a trivial manner, and further rows are for different continual learning strategies which attempt to dampen the amount of forgetting. Reported is the Dice of the final model after training in the orders $UCL \rightarrow I2CVB \rightarrow ISBI \rightarrow DecathProst$ (prostate), $HarP \rightarrow Dryad \rightarrow DecathHip$ (hippocampus) and $Siemens \rightarrow Philips$ (cardiac).

**Figure 3:**
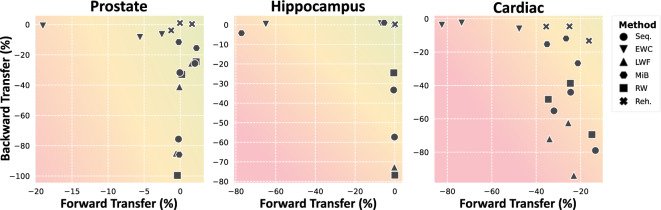
Relative backward (y-axis) and forward (x-axis) transfer for the continual segmentation of three use cases, averaged over all the respective data. Each marker corresponds to a training stage and anatomic stucture. Backward transfer is the *inverse* forgetting and forward transfer measures how well the model adapts to future tasks. For both metrics, higher is better, and results near zero can be realistically expected.

Over all anatomies, the *Rehearsal*^29^ (Reh.) method is effective at preventing forgetting. This is consistent with previous research^29^. However, this strategy cannot always be used as it requires samples to be stored from previous tasks in order to interleave them in future training. This is not possible in many scenarios, where rehearsal would be an additional upper bound. In these cases, *EWC* and *MiB* reliably reduce the amount of forgetting in early tasks. In contrast, *LwF* and *RW* do not seem to translate well to the task of semantic segmentation. We directly illustrate the forgetting as inverse *backward transfer* in Fig. 3 (y-axis), where we see that *EWC* (▼), *MiB* () and *Rehearsal* (✖) maintain high backward transfer scores.

Note, however, that this often comes at the cost of a loss of model plasticity, reducing the performance on later tasks. For instance, while the sequential model shows a Dice of 91.91% in *DecathProst* (the last task), it decreases to 87.79% for *EWC*. For hippocampus segmentation, this behavior is much more pronounced. The Dice on *DecathHip* falls from 90.92% to 31.93% for *EWC* and 20.75% for *MiB*. For the cardiac data, the performance deterioration on the initial *Siemens* task for *LwF* and *RW* and plasticity loss on *Philips* data for *EWC* and *MiB* are particularly noticeable for the more challenging myocardium (MI) and right ventricle (RV) classes. The loss of plasticity is illustrated as *forward transfer* (x-axis) in Fig. 3, where *EWC* shows negative values while *Rehearsal* remains close to zero.

**Figure 4:**
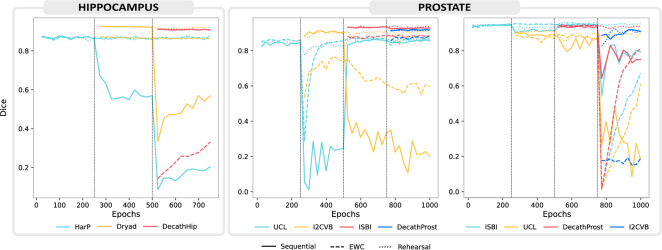
Learning trajectories for hippocampus and prostate segmentation, the last in two different orders, namely $UCL \rightarrow I2CVB \rightarrow ISBI \rightarrow DecathProst$ and $ISBI \rightarrow UCL \rightarrow DecathProst \rightarrow I2CVB$. The vertical lines mark task boundaries. Each tasks is displayed with a different color. We compare trivial *Sequential* training (solid lines) to *EWC* (dashed) and *Rehearsal* (dotted).

We further analyze the behavior of trivial sequential training alongside the best-performing *Rehearsal* method and *EWC* by observing the training trajectories in Fig. 4.

The solid lines for sequential training mostly depict a rapid fall after task boundaries. Both *Rehearsal* and *EWC* considerably reduce the amount of forgetting. However, the decreased plasticity manifesting as a negative forward transfer for *EWC* is evident, with the dashed lines of a new task often starting below the sequential equivalents most notably in Fig. 4 for *DecathHip* at the third hippocampus stage.  

For the prostate experiments in the order $UCL \rightarrow I2CVB \rightarrow ISBI \rightarrow DecathProst$, we notice an unexpected recovery for *UCL* (cyan) after training with *I2CVB* (second stage) is concluded. However, this is likely due to the inherent good performance of models trained with *ISBI* and *DecathProst* on *UCL* (see Fig. 2).

we repeat the experiment in the oder $ISBI \rightarrow UCL \rightarrow DecathProst \rightarrow I2CVB$, we see a behaviour more similar to that observed for the hippocampus, where there is a continual deterioration of performance for older tasks and a lost of model plasticity for *EWC* manifested in a low starting performance for the last task, I2CVB.

This shows how important task orderings are when comparing continual learning methods. Ideally, all orderings should be considered, but this can be computationally prohibiting when training 3-dimensional segmentation architectures. Alternatively, static in-distribution and inter-task performance results should be taken into account. Nevertheless, this can only be done for retrospective studies. Prospectively, the order of the tasks is given, and the user must train with tasks as they become available, without any knowledge on how related these are to data that will become available later on.

### Tuning the plasticity/rigidity trade-off

Most continual learning methods allow the tuning of model rigidity through some hyperparameters. For instance, the *EWC* $\lambda$ decides how much the divergence from previous model states should be penalized. A larger $\lambda$ prioritizes knowledge preservation whereas a smaller $\lambda$ allows the model to adapt more easily to the new distribution.

Unfortunately, unlike in static training settings where hyperparameters can be tuned with a validation set, in a real continual setting we have no access to samples from previous tasks and no information on which data the model will receive later on. It is therefore extremely difficult to decide on good hyperparameters, and we must follow the guidelines in the literature, preliminarly observe the loss trajectories in the current task or guide our settings through the results of other experiments. That is the strategy we follow in this work.

In Table 2, we include retrospective results across three hyperparameter setting for each continual learning method. These include the default settings used in Table 1 ($\lambda = 0.4$ for *EWC*, $T = 2$ for *LwF*, $\alpha =0.9$ for *MiB* and $\lambda = 0.4$ for *RW*) and others that we deemed reasonable after analyzing those results. Particularly, we notice that a lower $\lambda$ for *EWC* and lower $\alpha$ for *MiB* are beneficial, allowing for more model plasticity while still preserving knowledge. Other settings did not improve results for *LwF* or *RW*.

We highlight that this does not translate to higher performance on the use cases of *prostate* and *hippocampus*. In fact, though *EWC* and *MiB* lower the plasticity of the models as seen in Table 1, they are not *too rigid*, as we also notice some forgetting (e.g. for *ISBI*, *HarP* and *Dryad*). Setting hyperparameters for real deployment or prospective studies is extremely challenging and one problem when applying continual learning methodologies in real dynamic settings, as a rigidity/plasticity trade-off cannot be reliably selected by observing the results on other use cases.

### Multi-head architectures

In previous experiments, we assumed that the entire model was sequentially trained. Continual learning is sometimes evaluated in a *multi-head* setting where the last network layer is kept task-dependent and not updated after training with its respective task^15^. During inference, the corresponding head is used alongside the shared body. Further, there are two alternatives in terms of body updating: the body can remain *plastic* and thus be updated as time goes on or be *frozen* after the first training stage.

If the task precedence is not known for a sample during inference, it can be inferred from image characteristics such as the distribution of intensity values or the ability of an autoencoder to reconstruct it^19,30^. In this work, we assume that this information is available.

**Figure 5:**
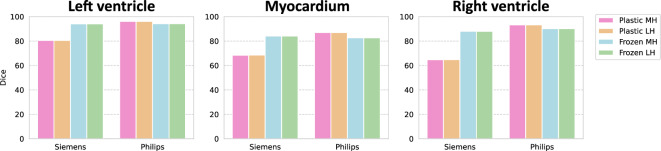
Four settings for training and constructing a model: allowing the model to adapt and keeping task-dependent heads (Plastic MH, pink) or sharing one head (Plastic LH, orange), freezing the body after the first task and keeping task-dependent heads (Frozen MH, light blue) or sharing one head (Frozen LH, green). The Dice is reported for three cardiac structures.

In Fig. 5, we explore the four possibilities of training vs. freezing the shared body and maintaining one vs. task-independent heads. We observe that the difference between maintaining one vs. separate heads (and selecting the appropriate one during inference) is minimal. In contrast, the practice of freezing the body prevents forgetting on the early *Siemens* task, though at the cost of slightly lower performance on the second *Philips* task (as would be expected due to plasticity loss). Looking at Fig. 2, the model trained only on *Siemens* data performs quite well on *Philips*. This indicates that the loss of plasticity could have a greater effect on the performance for a different data corpus.

### Qualitative evaluation

**Figure 6:**
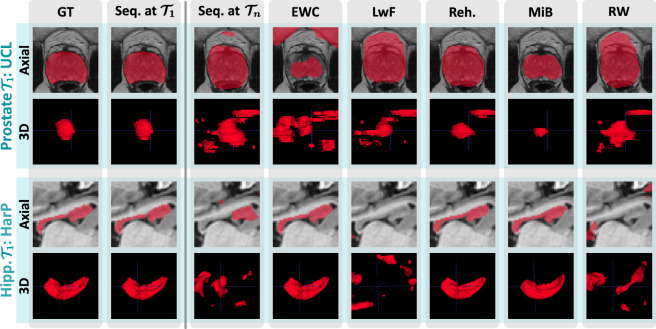
Qualitative deterioration of segmentation performance when training models sequentially for *UCL* and *HarP*, for which we display region-of-interest crops of axial views and 3D renderings produced with *ITK-SNAP*^31^.

In the following, we illustrate visually how continual learning affects the integrity of the segmentation masks. Unlike image classification, segmentations may give a direct indication of *when* and *how* a model is failing. Figure 6 displays examples from the *UCL* and *HarP* datasets, which are the first tasks for the prostate and hippocampus use cases, respectively.

The first and second columns show the ground truth and the segmentation produced by the model right after finishing training with the corresponding task. Further columns show the prediction of the final model with different continual learning strategies. As when trivially training the model in a sequential fashion (Seq. at $\mathscr {T}_n$), methods *LwF* and *RW* produce scattered segmentation masks with additional connected components. *EWC* maintains the integrity of the hippocampus segmentation, but not the prostate one. This is likely due to the increased rigidity of the hippocampus model, which in turn results in negative forward transfer (see Fig. 3). *Rehearsal* generally maintains the correct shapes, though the prostate mask is larger than should be and includes one additional connected component. Finally, *MiB* successfully produces reasonable masks in both cases, though slightly lower-segments the prostate.

### Hardware and run times

Our experiments were carried out in a system with 8 NVIDIA Tesla T4 (16 GB) GPUs, 2 Intel Xeon Silver 4210 CPUs, and 256 GB DDR4 RAM. Experiments were run in parallel, each taking up one GPU with the exception of the LwF experiments for the prostate use case, where 2 GPUs were used in tandem.

Table 3 provides an overview of the training times needed for one epoch for each method and anatomy. The hippocampus experiments were the fastest due to the lower resolution. *MiB* requires significantly more time that sequential training, and the duration of a *LwF* epoch increases heavily as the length of the task sequence grows (noticeable for the four-task prostate experiments), even as part of the network is frozen.

**Table 3:** Seconds required for completing one epoch of training. We report the mean and standard deviation over each anatomy, and in the case of the cardiac experiments, over both training orders.

	Prostate	Hippocampus	Cardiac
Seq.	214.3 (± 3.1)	117.3 (± 20.6)	194.5 (± 24.6)
EWC	215.25 (± 3.9)	131.0 (± 5.2)	197.3 (± 24.1)
LwF	423.3 (± 591.5)	233.3 (± 252.2)	174.7 (± 87.3)
MiB	365.5 (± 99.1)	212.0 (± 72.0)	281.5 (± 90.5)
RW	223.5 (± 1.3)	136.1 (± 1.0)	202.3 (± 26.0)
Reh.	206.0 (± 3.16)	140.3 (± 21.6)	195.5 (± 22.6)

Particularly for medical image segmentation, where hardware requirements are significant and potentially prohibitive, the computational overhead should be considered when selecting a continual learning strategy. In particular, it may be wise to avoid methods that increase the duration of each epoch linearly with the length of the task sequence.

## Discussion

In dynamic clinical environments, models are needed that can adapt to changing imaging protocols and disease patterns. While the importance of continual learning for medical imaging segmentation is being recognized, our community lacks the reporting standards and benchmark datasets that researchers employ for natural image classification.

With the *Lifelong nnU-Net*, we *establish a framework for the standardized evaluation of continual segmentation*. We extend the popular *nnU-Net* pipeline with all components needed for training and evaluating segmentation architectures in a sequential fashion, including five popular continual learning strategies and metrics specific to continual paradigms.

Our evaluation across three different segmentation use cases allows us to gain valuable insights. Consistent with previous research^29^, *Rehearsal* leads to the best results, considerably decreasing forgetting by interleaving a subset of cases from previous tasks in the training data. In our experiments, we interleave a fixed percentage of the past training data, but many strategies exist for optimizing the memory buffer or replicating cases when some tasks are under-represented. Of course, a rehearsal-based strategy is only feasible if this data can be stored. For scenarios where this is not the case due to patient privacy considerations, the *EWC* and *MiB* methods prove to be suitable alternatives, effectively reducing forgetting though at the cost of reducing the ability of the model to adapt to new tasks. Finally, the *LwF* and *RW* methods do not appear to be well-suited to our setup. Though they could be further tuned to allow for more knowledge preservation in retrospective experiments, this is not feasible during actual deployment, as model deterioration on previous tasks cannot be measured.

One disappointing takeaway in our study is that *no method resulted in positive backward transfer (BWT)*. This is clearly illustrated in Fig. 3, where we see that even the best methods only manage to prevent forgetting, reaching a BWT of zero. This means that *no knowledge acquired from later tasks improves performance on earlier tasks*. Therefore, maintaining wholly independent models and using the corresponding model during inference would outperform all explored continual learning methods. We also *only saw positive forward transfer in the prostate experiments*. This means that preceding training with earlier tasks and then fine-tuning only minimally improves performance when compared to training a model with the corresponding task from scratch.

In addition, we found that the practice of maintaining task-specific heads, common in the continual learning literature, *do not significantly affect the performance for continual segmentation in medical images*. This is the case both when the body is frozen after the first stage just as when it remains plastic. Further studies should look into leaving a greater portion of the network task-specific.

We have identified several limitations in our study. Firstly, we limited our study to the full-resolution patch-based 3D nnU-Net variant, which is suggested for most applications. We did not repeat our experiments on the slice-by-slice or 3D down-sampled networks. Our evaluation also focuses on the *incremental domain learning* scenario which is most relevant in the context of medical imaging^8^.

Secondly, as of now, there is a limited catalog of continual learning methods in the *Lifelong nnU-Net* framework. We looked to have sufficient representation of individual approaches across different strategies, and implemented a mixture of highly popular but older methods (simple *Rehearsal*, *EWC* and *LwF*) and newer approaches (*MiB* and *RW*). In the future, we hope this catalog grows both from our efforts and the contributions of other members of the community.

## Methods

An effective framework for continual image segmentation has the following requirements:

1. It has all components for achieving high-quality static segmentation results and supports both two- and three-dimensional architectures (like the *nnU-Net*),
2. Simplifies the evaluation of incremental domain scenarios by relying on widely accepted dataset formats and the alignment of label characteristics across datasets,
3. Includes integrated evaluation logic that tracks the performance of the model for different tasks during training with appropriate metrics, and
4. Supports existing state-of-the-art continual learning solutions, including the training of *multi-head models* that maintain both *shared* and *task-independent* parameters.

We start this section by introducing the three segmentation use cases that we explore, as well as our notation. We then outline how we approach each one of the requirements stated above to ensure that the *Lifelong nnU-Net* framework provides a solid foundation for medical continual learning research. Finally, we describe the continual learning methods used and briefly state details of our experimental setup.

### Datasets

We explore the problem of continual image segmentation for three very different use cases. To ensure reproducibility, we use only openly available datasets and align the label characteristics according to the process outlined below. For each anatomy, we select an array of datasets that act as our *tasks* $\mathscr {T}_1 ... \mathscr {T}_n$. Table 4 provides an overview of data and label characteristics for all datasets.

**Table 4:** Image and label characteristics; including the number of cases, mean resolution and spacing, and mean percentage of voxels labeled as the region-of-interest (ROI).

Dataset	# Cases	Resolution	Spacing	ROI %
UCL	13	[24 384 384]	[3.3 0.5 0.5]	0.01
I2CVB	19	[64 384 384]	[1.3 0.5 0.4]	0.01
ISBI	30	[19 384 384]	[3.7 0.5 0.5]	0.03
DecathProst	32	[19 316 316]	[1.0 1.0 1.0]	0.03
HarP	270	[48 64 64]	[1.0 1.0 1.0]	0.01
Dryad	50	[48 64 64]	[1.0 1.0 1.0]	0.02
DecathHip	260	[36 50 35]	[1.0 1.0 1.0]	0.05
Siemens	75	[12 239 209]	[1.3 1.3 9.2]	0.02/0.01/0.02
Philips	75	[11 307 307]	[1.3 1.3 9.9]	0.01/0.01/0.01

**Figure 7:**
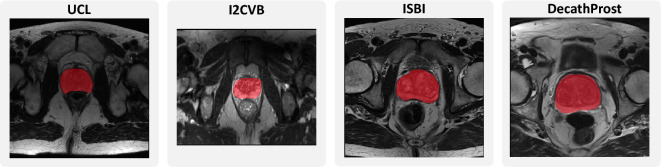
Exemplary slices for four subjects from the prostate segmentation datasets.

The first use case we approach is the segmentation of the prostate in T2-weighted MRIs, for which we use a corpus of four data sources. We utilize the data as provided in the *Multi-site Dataset for Prostate MRI Segmentation Challenge*^32,33^ for sites A (*ISBI*^34^), C (*I2CVB*^35^) and D (*UCL*^36^). Lastly, we use the data provided as part of the *Medical Segmentation Decathlon*^37^ (*DecathProst*). Some segmentation masks contain two labels representing the peripheral zone and central gland, which we join into one *prostate* label to ensure consistency across the corpus. Prostate segmentation is a rather easy problem, though crucial for determining the possible location of tumorous tissue preceding a biopsy, and the shape of the prostate varies very little between different patients. Figure 7 shows examples of the four datasets.

**Figure 8:**
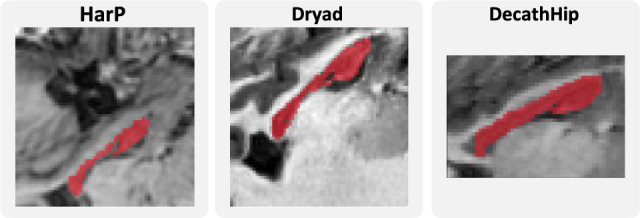
Exemplary slices for three subjects from the hippocampus segmentation datasets.

The second is the segmentation of the hippocampus in T1-weighted MRIs, for which we include three data sources. The *Harmonized Hippocampal Protocol* data^38^, henceforth referred to as *HarP*, contains senior healthy subjects and patients with Alzheimer’s disease. The *Dryad*^39^ dataset has fifty additional healthy patients. As a third data source, we use the images provided as part of the *Medical Segmentation Decathlon*^37^ (*DecathHip*), from both healthy adults and schizophrenia patients. For the segmentation of the hippocampus, Dices of over 90% can be expected^24^. Exemplary image slices from all three datasets can be found in Fig. 8.

**Figure 9:**
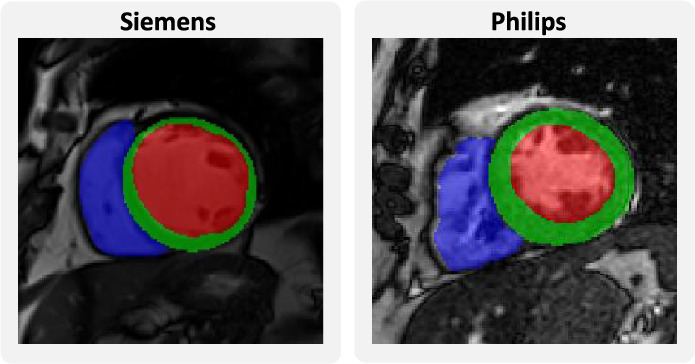
Exemplary slices for subjects from the two cardiac datasets. Segmented are the left ventricle (red), myocardium (green), and right ventricle (blue).

Finally, we explore the segmentation of the left and right ventricles (LV and RV) and the myocardium (MI) in cardiac MRIs. We make use of the data released for the *Multi-Centre, Multi-Vendor & Multi-Disease Cardiac Image Segmentation Challenge (M &Ms)*^40^, which includes 75 labeled cases acquired with Siemens and 75 cases acquired with Philips scanners. This entails the additional difficulty of being a multi-class problem, which allows us to observe how the performance of different anatomical structures varies depending on the shape and size of the region of interest. Exemplary slices can be observed in Fig. 9.

We select these three problem settings to ensure variability across modality, shape and size of the segmentation masks, and difficulty of the task at hand. Of course, our framework allows for the fast evaluation of further use cases. For all datasets, we divide 20% of the data for test purposes and maintain this split across all experiments. *We make the splits publicly available alongside our code.*

### Notation

Consider *n* tasks $\mathscr {T}_1$, ..., $\mathscr {T}_n$. Model $\mathscr {F}_2$ is trained only on the training data of task $\mathscr {T}_2$. Model $\mathscr {F}_{[1, 2, 3]}$ was trained sequentially on tasks $\mathscr {T}_1$, $\mathscr {T}_2$ and $\mathscr {T}_3$, in that order. $\mathscr {F}_{\left\{ 1, 2, 3 \right\} }$ is instead a *static* model, trained with shuffled training data from all three tasks. Finally, we use $\mathscr {F}_i(\mathscr {T}_j)$ to refer to the performance of model $\mathscr {F}_i$ applied to the test data of task $\mathscr {T}_j$.

### Aligning label characteristics

Very often, segmentation datasets that explore similar problems are not uniform in terms of label structure. Continual learning is only feasible if the annotations are consistent throughout datasets. Therefore, before a model can be trained in a continual fashion, a crucial pre-processing step involves *aligning label characteristics*.

**Figure 10:**
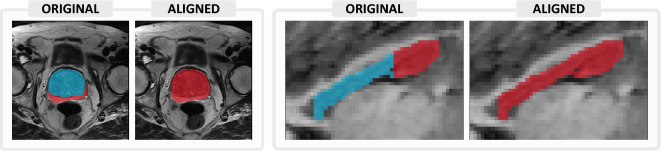
Alignment of label characteristics for prostate (merging the central gland and peripheral zone) and hippocampus (merging head and body).

Consider, for instance, the problem of prostate segmentation. Dataset $\mathscr {T}_1$ may include annotations for the *prostate* class, distinguishing prostate voxels (which take value 1 in the segmentation mask) from the background marked with zeros. Dataset $\mathscr {T}_2$ may instead include annotations for the central gland (label 1) and peripheral zone (label 2), two regions that together make up the prostate. Yet another dataset, $\mathscr {T}_3$, may include annotations for both the prostate (label 1) and bladder (label 2). We can align these labels to take up the structure of dataset *A* by converting annotations for labels 1 and 2 to class 1 (prostate) in dataset *B* and converting label 2 (bladder) to class 0 (background) for dataset *C*. This process is visualized in Fig. 10. Of course, an alternative scenario would be *incremental label learning*, where the number of labels grows over time. In this case, one would maintain the separate *bladder* label in $\mathscr {T}_3$.

Aligning these characteristics is crucial for obtaining enough open-source data for a meaningful evaluation of different use cases. In *Lifelong nnU-Net*, we have included a pre-processing script that easily performs these steps.

### Multi-head models

The natural alternative to training a model sequentially—under our data availability constraints—is maintaining one model per task and selecting which model to use for each subject during inference. This option ensures that *no forgetting occurs*, though it leaves out any possibility for backward and forward transfer and increases the memory requirements linearly with the number of tasks. Several continual learning methods adopt an intermediate approach: earlier layers are shared but the last layers are kept task-specific^25,26^. The intuition is that *multi-head* models allow earlier parameters to learn from new data while the last network layers conserve task-specific information.

**Figure 11:**
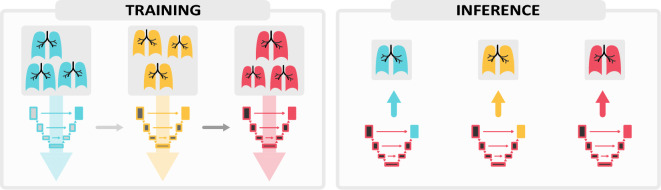
During training, the shared body is sequentially modified while the model *head* remains task-specific. During inference, the corresponding head is merged with the final state of the shared body to extract a prediction.

We implement this behavior in the *Lifelong nnU-Net* framework as visualized in Fig. 11. For the first task, the training proceeds as usual. Before training takes place with the second task, the model head is replicated. Training then goes on with the shared body and the new head. This process is repeated for all tasks. During inference, a head is selected for each image and combined with the shared body. Additionally, we include the option of freezing the shared body after the first training stage, and only updating the head(s). Which parameters make up the *head* is determined by the user. For the experiments on multi-head architectures, we use *seg_outputs* as a split point.

### Evaluation logic and metrics

The nnU-Net includes methods for dataset preparation, training and performing inference. The performance on a validation set is monitored with the *Dice coefficient*, which measures the intersection of voxels segmented for a class in the prediction *A* and the ground truth delineation *B*, normalized by the total number of voxels in that class.

1 $$\begin{aligned} {Dice = \frac{2|A\cap B|}{|A|+|B|}} \end{aligned}$$

Considering the requirements of continual learning, we expand on this logic with:

- An *evaluation module* for testing on all datasets of interest, to be run after training has concluded, and
- The extended behavior of tracking the performance during training *on several different validation sets*. This gives the user insight into how the training with any task $\mathscr {T}_i$ gradually affects the training with task $\mathscr {T}_j$, and allows them to export expressive training trajectories as that visualized in Fig. 4.

These modifications allow for quick validation of continual learning settings and simplify the validation on out-of-distribution data without needing to store all model states.

In addition to observing the segmentation performance in the form of the Dice coefficient , we explore metrics from continual learning research that provide a more intuitive way of understanding the results.

The primary goal of continual learning in the open world, where distribution shifts are commonplace, is to avoid overfitting to image characteristics in the last batches so that the final model can cope with samples from all seen sources. Besides avoiding the dreaded *catastrophic forgetting*, the model should ideally achieve *both backward and forward transfer*^41^ and ensure reliable performance across all subject groups.

#### Forgetting and backward transfer (BWT)

we measure the difference between the performance of a model in task $\mathscr {T}_i$ right after training with that task and after training with further tasks. If the result is negative, this implies *forgetting* has occurred. If, instead, the result is positive, then the desirable property of *backward transfer* was achieved, e.g. training with tasks $\mathscr {T}_{i+1}$ improves the performance on task $\mathscr {T}_{i}$.

2 $$\begin{aligned} BWT=\mathscr {F}_{[..., i, ...]}(\mathscr {T}_i) - \mathscr {F}_{[..., i]}(\mathscr {T}_i) \end{aligned}$$

#### Forward transfer (FWT)

we calculate how advantageous the fine-tuning process is for a certain task, i.e. the difference between the continual model state right after training with task $\mathscr {T}_i$ and model $\mathscr {F}_i$ trained solely on task $\mathscr {T}_i$. A positive result implies that preceding training with data from other tasks improves the performance of the model after fine-tuning, and a negative result signifies that the model is unable to adapt to $\mathscr {T}_i$. This second case may occur when using certain continual learning methods that reduce model plasticity. Though other definitions consider this metric for all future tasks, we focus on the corresponding task and define:

3 $$\begin{aligned} FWT=\mathscr {F}_{[..., i]}(\mathscr {T}_i) - \mathscr {F}_{i}(\mathscr {T}_i) \end{aligned}$$

For both metrics, we report the *relative* performance change with respect to the right-hand side of the subtraction. This allows us to compare the performance across anatomies with different segmentation difficulties.

#### Inter-task performance

We train one separate model for each task and visualize how each model performs on the other tasks (see Fig. 2). This helps us estimate the compatibility between tasks, which should facilitate continual learning.

### Continual learning methods

We hereby briefly describe the methods we compare in this work. We refer the reader to our code base and documentation for further details on the implementation.

#### Rehearsal

**Figure 12:**
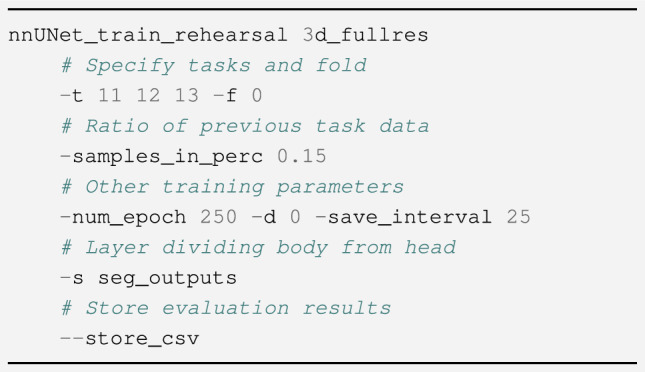
Command-line directive for performing training with rehearsal. An optional *seed* argument can also be used to select samples from previous tasks in a deterministic manner.

The simplest form of lifelong learning entails interleaving samples from previous tasks into the training data. The size of the *memory buffer* determines how many of such samples are stored. The *Lifelong nnU-Net* framework allows the user to perform this type of training *with only one line of code*, specifying the tasks and size of the memory buffer. The necessary command is exemplified in Fig. 12. Rehearsal is a very effective strategy that consistently ensures good performance, though not admissible in settings that do not allow the storage of training samples.

Running other methods proceeds in a similar manner, although with different hyperparameters.

#### Elastic Weight Consolidation

Regularization-based approaches assess the *importance* of each training parameter and penalize the divergence from the previous state weighted by the importance. The $\lambda$ parameter weights the magnitude of this regularization loss and the target loss (in our case for image segmentation). The main difference among regularization-based methods consists in how the importance is calculated. The popular *EWC* method^25^ utilizes the *Fisher Information Matrix*, which measures how far model outputs are from the one-hot encoded predictions.

#### Learning without forgetting

The *LwF* method^26^ consists of three training stages. (1) After the training phase for task $\mathscr {T}_{i}$, and before starting task $\mathscr {T}_{i+1}$, model outputs $\mathscr {F}_{[i]}^i(\mathscr {T}_{i+1})$ are recorded and a new head is created for $\mathscr {T}_{i+1}$. (2) Then, shared parameters are frozen and only the new head is trained. (3) Finally, the shared body alongside all heads is fine-tuned. The outputs recorded in the first step are used for training previous heads.

#### Riemannian walk

A combination of the previously introduced *EWC* with *Path Integral* forms *RW*^27^. The main difference to *EWC* is the online calculation of the Fisher Information Matrix for assessing the importance of each parameter. With this modification, the additional forward pass at the end of the training to obtain the Fisher values can be omitted.

#### Modeling the background

The *MiB*^28^ method—specifically developed for semantic segmentation—uses a modified cross entropy loss in combination with a knowledge distillation term. The knowledge distillation is used to force the activation of the current network $\mathscr {F}_{\theta }$ to be similar to the previous network $\mathscr {F}_{\theta _{i-1}}$.

### Experimental details and hyperparameters

We train the full-resolution version of the nnU-Net which is recommended for most applications^24^. This is a patch-based, three dimensional network. For each of our three use cases, models are trained with every dataset for 250 epochs.

The nnU-Net automatically configures hyperparameters for the network architecture and training process—such as the number of encoding blocks, learning rate and patch size—from the training data. It is possible that these parameters differ between datasets of the same use case. In our framework, we always use the configuration chosen for the *first dataset*, which is the most realistic choice as in a real continual setting only this data is available when building the architecture.

Unless otherwise stated, we select hyperparameters used in previous work or which showed reasonable loss trajectories in preliminary experiments with a fraction of the epochs. For the cardiac experiment, we test several settings in Table 2. For *Rehearsal*, we state the number of cases from previously seen tasks to be included in the current task to 25%. For *EWC*, we use the default value of $\lambda =0.4$ to weigh the regularization term. In the case of *LwF*, we set the knowledge distillation temperature to 8 for hippocampus and 64 for prostate. For *RW*, $\lambda =0.4$ for regularization and $\alpha =0.9$ for calculating the Fisher values are used. *MiB* *hardifies* the soft labels with $\alpha =0.9$ for hippocampus and $\alpha =0.75$ for prostate.

We refer the reader to our code base and documentation for further details.

## Data Availability

All datasets used in this work are openly available and downloading instructions can be found under the respective references.

## Code availability

Our code is available under https://github.com/MECLabTUDA/Lifelong-nnUNet. Upon request, we can facilitate access to the trained models.
